# Aspirin regulation of c-myc and cyclinD1 proteins to overcome tamoxifen resistance in estrogen receptor-positive breast cancer cells

**DOI:** 10.18632/oncotarget.16325

**Published:** 2017-03-17

**Authors:** Ran Cheng, Ya-Jing Liu, Jun-Wei Cui, Man Yang, Xiao-Ling Liu, Peng Li, Zhan Wang, Li-Zhang Zhu, Si-Yi Lu, Li Zou, Xiao-Qin Wu, Yu-Xia Li, You Zhou, Zheng-Yu Fang, Wei Wei

**Affiliations:** ^1^ Department of Breast Surgery, Peking University Shenzhen Hospital, Shenzhen, 518036, China; ^2^ Institute of Biomedical Research, Shenzhen PKU-HKUST Medical Center, Shenzhen, 518036, China

**Keywords:** aspirin, ER-positive breast cancer, c-myc, cyclinD1, tamoxifen resistance

## Abstract

Tamoxifen is still the most commonly used endocrine therapy drug for estrogen receptor (ER)-positive breast cancer patients and has an excellent outcome, but tamoxifen resistance remains a great impediment to successful treatment. Recent studies have prompted an anti-tumor effect of aspirin. Here, we demonstrated that aspirin not only inhibits the growth of ER-positive breast cancer cell line MCF-7, especially when combined with tamoxifen, but also has a potential function to overcome tamoxifen resistance in MCF-7/TAM. Aspirin combined with tamoxifen can down regulate cyclinD1 and block cell cycle in G0/G1 phase. Besides, tamoxifen alone represses c-myc, progesterone receptor (PR) and cyclinD1 in MCF-7 cell line but not in MCF-7/TAM, while aspirin combined with tamoxifen can inhibit the expression of these proteins in the resistant cell line. When knocking down c-myc in MCF-7/TAM, cells become more sensitive to tamoxifen, cell cycle is blocked as well, indicating that aspirin can regulate c-myc and cyclinD1 proteins to overcome tamoxifen resistance. Our study discovered a novel role of aspirin based on its anti-tumor effect, and put forward some kinds of possible mechanisms of tamoxifen resistance in ER-positive breast cancer cells, providing a new strategy for the treatment of ER-positive breast carcinoma.

## INTRODUCTION

Approximately 50%–70% of breast cancers are considered estrogen receptor (ER) positive [1]. Estrogen signaling plays a central role in ER-positive breast cancer, which involves in cell proliferation and survival [1, 2]. Tamoxifen, a selective estrogen receptor modulator (SERM), has been the most widely used endocrine treatment in ER positive breast cancer patients for more than 30 years [3, 4]. However, primary or acquired resistance to tamoxifen results in therapeutic failure, which needs to be solved in the clinical application [5–8].

Researches show that tamoxifen can block cell cycle and inhibit cell proliferation in sensitive breast cancer cells [9–11]. But when there exists drug resistance, tamoxifen cannot play a role [12]. Generally, cyclin-dependent kinases (CDKs) involve in the cell cycle phase transitions and also boost gene transcription in mammals [13]. More literatures suggest that cyclin-dependent kinases (especially, CDK4 and CDK6) have been identified as the major oncogenic drivers among CDKs in the cell cycle transitions [14, 15]. Evidence indicates that cyclinD-CDK4/CDK6 axis has an important role in breast cancer cell survival and proliferation [14–18]. Aberrant regulation of cyclinD1 leads to tamoxifen resistance, so drugs targeting the axis can be used in the treatment of breast cancer [17, 19], which might be also applicable in patients with advanced breast cancer who are resistant to endocrine treatment.

A considerable body of evidence strongly suggests that non-steroidal anti-inflammatory drugs (NSAIDs) such as aspirin can reduce the risk of cancers associated with colon, breast, gastric, prostate, lung, and skin [20–25]. But the exact mechanisms of aspirin that exert its anti-tumor effect are yet to be elucidated [21, 26]. Studies have reported that aspirin and its primary metabolite salicylic acid both have the ability to decrease the mRNA and protein levels of c-myc in human colon cancer cell lines, which might be one of the mechanisms for the antineoplastic activity [27, 28]. Recently, Dachineni *et al* [29] have shown that aspirin and salicylic acid can down-regulate a number of cyclins and cyclin dependent kinases (CDKs) in multiple cancer cell lines, which collectively suggests that inhibitory effect may occur through down-regulation of these cell cycle regulatory proteins, providing a novel mechanism for the anti-tumor effect of aspirin and salicylic.

In our study, we used the ER-positive breast cancer cell line MCF-7 as the research model, which was sensitive to the anti-estrogen treatment. At the same time, tamoxifen resistant cell line MCF-7/TAM was used as the model to investigate the possible underlying molecular mechanism of tamoxifen resistance. According to our studies, there were some genes which may contribute to the tamoxifen resistance. A few studies have reported that *MYC* mRNA and c-myc protein can be inhibited by salicylates such as aspirin. This in turn reveals the anti-tumor effect of salicylates on colon cancer cell lines [27, 28]. So we attempted to use this drug in ER-positive breast cancer and combined it with the SERM 4-hydroxy-tamoxifen (4-OHT). Interestingly, aspirin not only had anti-tumor function on the two cell lines MCF-7 and MCF-7/TAM, but also restored the inhibitory effect of 4-OHT in tamoxifen resistant cell line MCF-7/TAM. Furthermore, we confirmed aspirin's anti-tumor function and potential role in overcoming tamoxifen resistance by blocking cell cycle. Then we found that aspirin down-regulated the tumor related protein cyclinD1, which was one of the key factors in the cyclinD-CDK4/CDK6 axis [13]. Aspirin combined with tamoxifen could block cell cycle in the G0/G1 phase in the two cell lines. Further, we knocked down the *MYC* gene and the effect of aspirin occurred. Our studies have discovered a novel role based on anti-tumor effect of aspirin, and have put forward a few possible mechanisms of tamoxifen resistance in ER-positive breast cancer cells, providing a new strategy for the treatment of ER-positive breast cancer.

## RESULTS

### Identification of MCF-7 and MCF-7/TAM, and comparison of the anti-tumor effect of 4-hydroxy-tamoxifen (4-OHT) on the two breast cancer cell lines

Through comparison with cell databases and identification of professional institutions, there was no deterioration caused by other human cells and there was no cytometaplasias observed in MCF-7 and MCF-7/TAM cell lines. Cell lines intended to be MCF-7 whose DNA was typing well.

**Figure 1:**
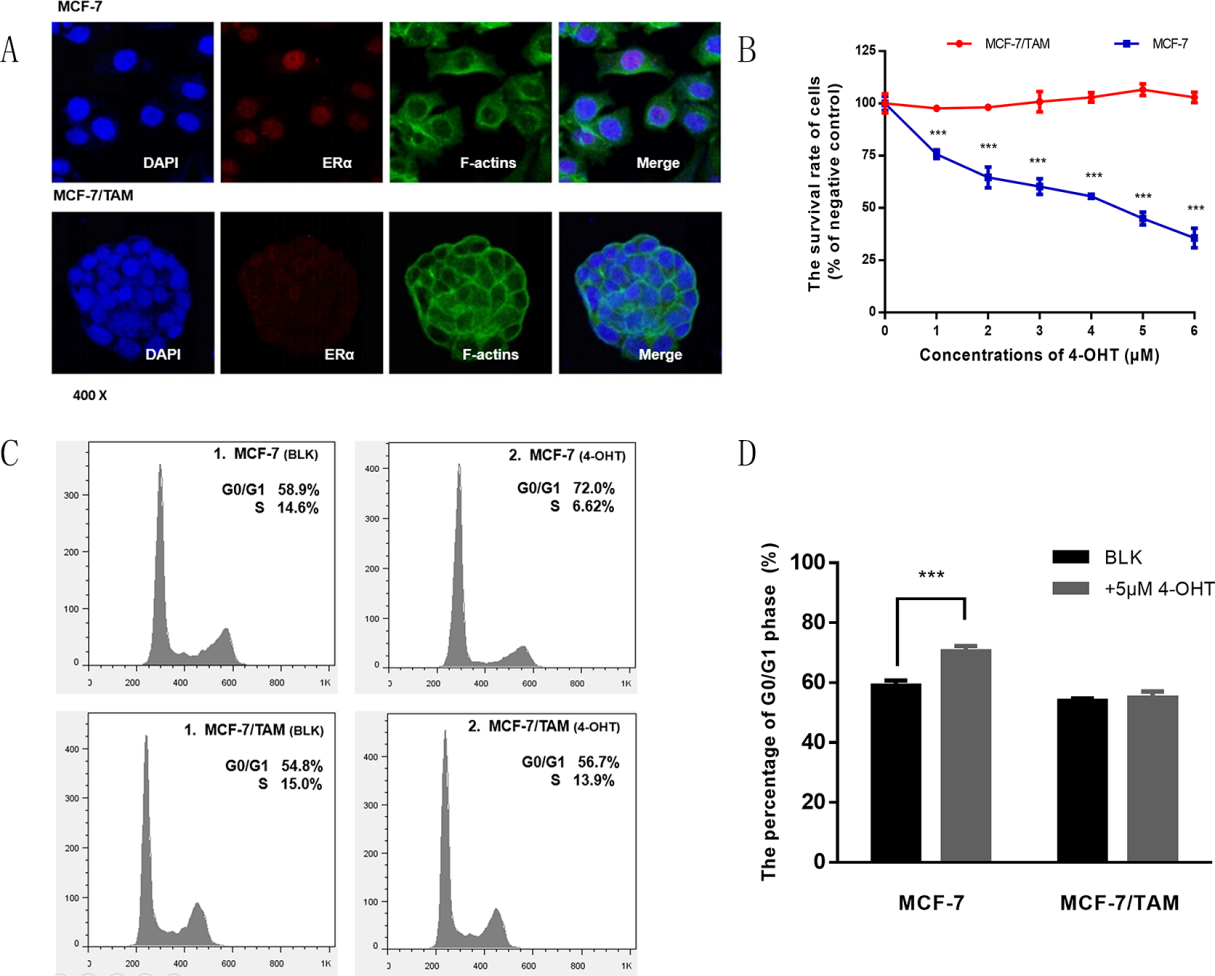
Detecting the expression of ER α and tamoxifen sensitivity in MCF-7 and MCF-7/TAM cell lines (**A**) The expression of ERα was observed using immunofluorescent assays in MCF-7 and MCF-7/TAM cell lines. (**B**) The survival rate of MCF-7 and MCF-7/TAM cells was tested by MTS Kit after treated with 4-OHT at the indicated concentrations (0–6 μM) (***p < 0.001). (**C**) MCF-7 and MCF-7/TAM cells were treated with 4-OHT (5 μM) for 72 h, then stained by PI and detected by flow cytometry analysis. (**D**) Bar chart represented the percentage of G0/G1 phase (***p < 0.001). All the experiments were repeated for at least three times. The results were presented as mean ± SEM.

Estrogen receptor α (ERα), the target of tamoxifen, is one of the most important biomarkers in MCF-7 cell line. Results from the immunofluorescent assay showed that ER*α* was expressed in every cell of both tamoxifen sensitive cell line MCF-7 and tamoxifen resistant cell line MCF-7/TAM, but the fluorescence intensity of MCF-7/TAM cells was much weaker than that of MCF-7 cells. Besides compared with MCF-7 cells, the morphology of MCF-7/TAM cells also changed, cells were smaller and more compact (Figure 1A).

To examine whether MCF-7 cells were sensitive to 4-OHT while MCF-7/TAM cells were resistant, we treated two cell lines with several concentrations of 4-OHT (0–6 μM) for 7 days and measured the cell survival rate compared with negative control. As shown in Figure 1B, there was an incremental inhibition effect of proliferation on MCF-7 cells but not on MCF-7/TAM cells, indicating that MCF-7/TAM cells were resistant to tamoxifen.

Previous studies suggest that tamoxifen can block cell cycle and inhibit cell proliferation in sensitive breast cancer cells [9–11]. Then we tested cell cycle by flow cytometry and found that the percentage of G0/G1 phase increased significantly in MCF-7 cells after treated with 4-OHT, but not in MCF-7/TAM cells (Figure 1C and 1D), which meant that cell cycle would be affected in MCF-7 cells, while in MCF-7/TAM cells there was no significant change of it.

### Aspirin has an obvious anti-tumor effect on both cell lines and a synergetic effect with tamoxifen on MCF-7/TAM

**Figure 2:**
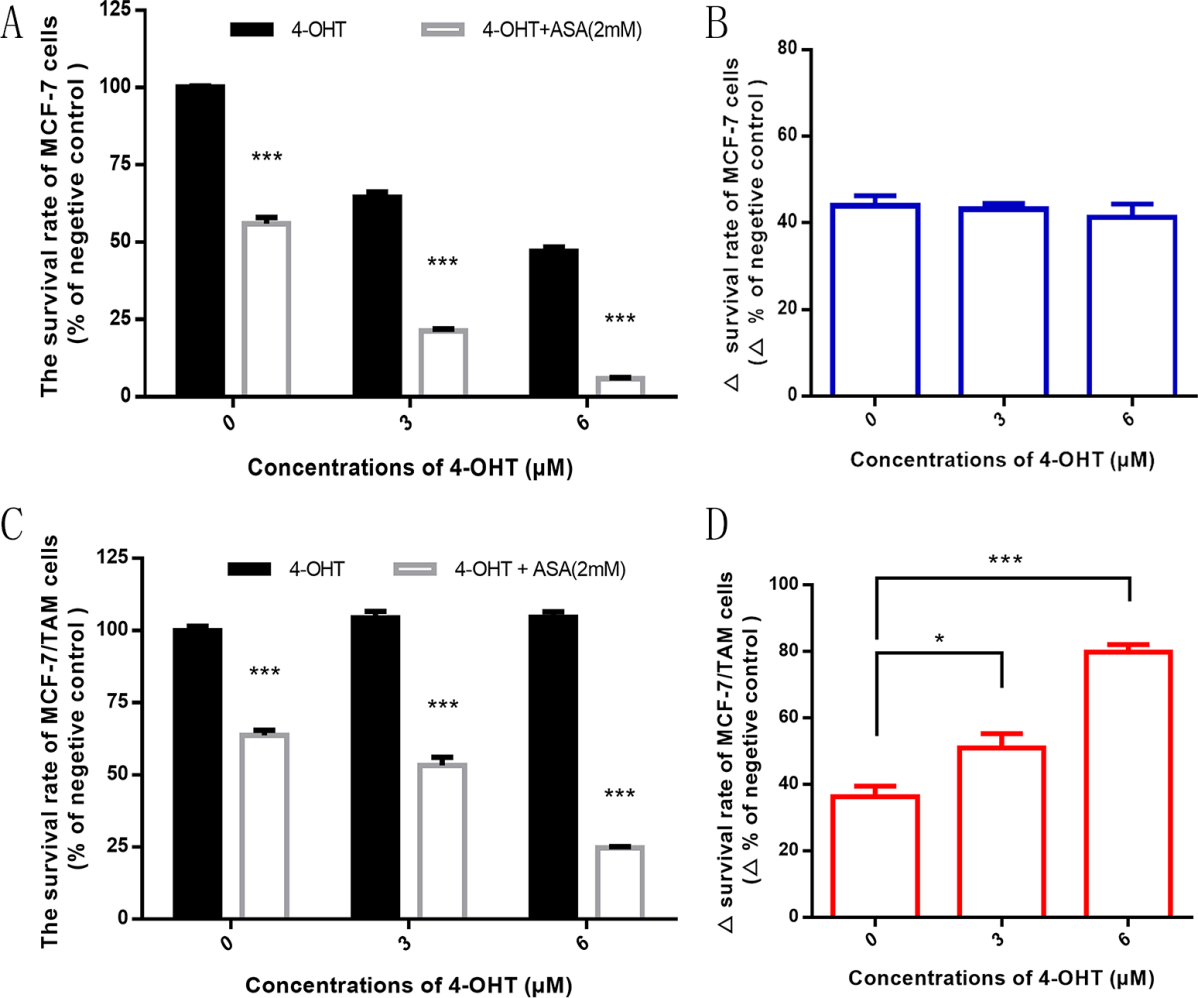
ASA has an obvious anti-tumor effect on both cell lines and can overcome tamoxifen resistance (**A**) After 7-day treatment, with the increasing concentrations of 4-OHT (0 μM, 3 μM and 6 μM), the survival rate of MCF-7 cells decreased and there was an additive inhibitory effect when combined with ASA (2 mM) (***p < 0.001). (**B**) The difference of survival rate between combined drug group and negative control (4-OHT alone) was observed at the indicated concentration of 4-OHT. There was no significant difference (*p* > 0.05). (**C**) After 7-day treatment, with the increasing concentrations of 4-OHT (0 μM, 3 μM and 6 μM), the survival rate of MCF-7/TAM cells decreased when combined with ASA (2 mM) (***p < 0.001), while there was no such phenomenon observed when using 4-OHT alone (*p >* 0.05). (**D**) The difference of survival rate between combined drug group and negative control (4-OHT alone) was observed at the indicated concentration of 4-OHT. There was significant difference observed (*p < 0.05 and ***p < 0.001). All the experiments were repeated for at least three times. The results were presented as mean ± SEM.

We used 4-OHT alone and combination with ASA to investigate the effect on two cell lines. As shown in Figure 2, the inhibitory effect of 4-OHT on MCF-7 and MCF-7/TAM cells was distinct. The survival rate of MCF-7 cells decreased along with the increase of concentrations of 4-OHT, and there was an additive inhibition observed when combined with 2 mM ASA (Figure 2A and 2B). These results suggested that ASA had anti-tumor effect on MCF-7 cell line.

We found that the survival rate of MCF-7/TAM cells decreased with the increasing concentrations of 4-OHT when combined with 2 mM ASA, while no such phenomenon was observed when using 4-OHT alone (Figure 2C). Also we figured out that at the same concentration of 4-OHT, there was difference of MCF-7/TAM cells survival rate between using 4-OHT alone and combined with 2 mM ASA. Meanwhile the difference significantly rose with the increase of concentrations of 4-OHT (Figure 2D), suggesting that ASA had an additional function to enhance the anti-tumor effect of tamoxifen on MCF-7/TAM cells. We deduced that ASA could not only have anti-tumor effect on ER-positive breast cancer cell lines, but also overcome tamoxifen resistance.

### ASA enhance the anti-tumor effect of 4-OHT through cell cycle arrest

**Figure 3:**
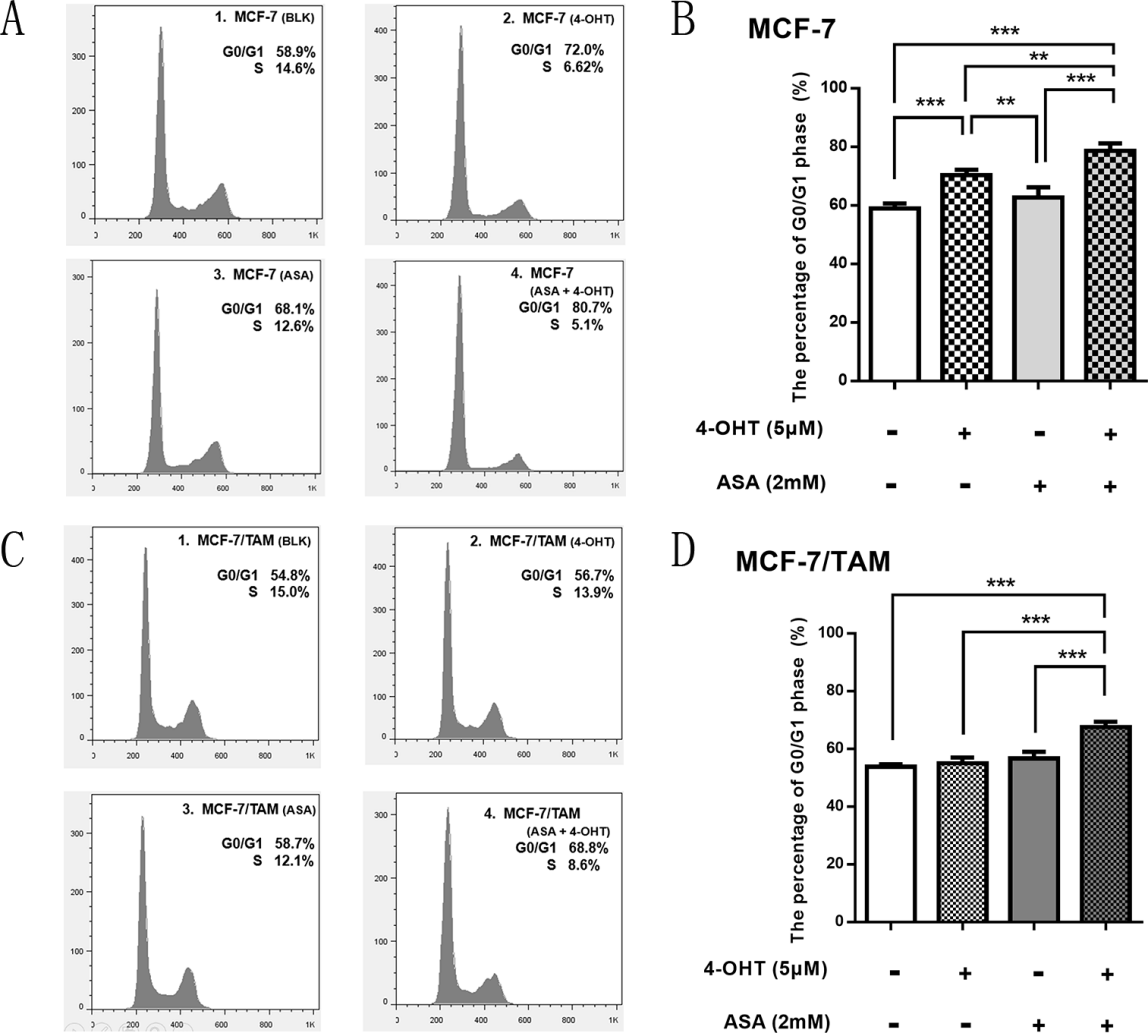
ASA enhance the anti-tumor effect of 4-OHT through cell cycle arrest (**A**) MCF-7 cells were respectively treated with 4-OHT (5 μM), ASA (2 mM), and 4-OHT (5 μM) combined with ASA (2 mM) for 72 h, then detected by flow cytometry analysis. (**B**) Bar charts represented the percentage of G0/G1 phase in different groups of MCF-7 cells. There was no significant difference observed between ASA group and negative control (*p >* 0.05), but significant difference was observed among other three groups (**p < 0.01; ***p < 0.001). (**C**) MCF-7/TAM cells were respectively treated with 4-OHT (5 μM), ASA (2 mM), and 4-OHT (5 μM) combined with ASA (2 mM) for 72 h, then detected by flow cytometry analysis. (**D**) Bar charts represented the percentage of G0/G1 phase in different groups of MCF-7/TAM cells. There was no significant difference among ASA group, 4-OHT group and negative control (*p >* 0.05), but when combining 4-OHT with ASA, the percentage of G0/G1 phase increased, which showed significant differences (***p < 0.001). All the experiments were repeated for at least three times. The results were presented as mean ± SEM.

As shown above, 4-OHT could block cell cycle in MCF-7 cells but not in MCF-7/TAM cells. We wondered whether there also existed changes of cell cycle in two cell lines when combining 4-OHT with 2 mM ASA. Then we tested by flow cytometry. As shown in Figure 3A and 3B, compared with negative control, the percentage of G0/G1 phase increased in the 4-OHT group and combination group in MCF-7 cell line, especially in the combination group, suggesting that cell cycle changed in different extent after treatment.

In MCF-7/TAM cell line, there was nearly no change of cell cycle among 4-OHT group, ASA group and negative control, but the percentage of G0/G1 phase increased evidently in the combination group (Figure 3C and 3D), which showed that more cells were arrested in G0/G1 phase when combining 4-OHT with ASA than using 4-OHT alone or ASA alone. Thus, we concluded that aspirin could enhance the cell-killing effect of 4-OHT through cell cycle arrest both in MCF-7 and MCF-7/TAM cell line. This might be a comparatively important mechanism of how ASA reverse 4-OHT resistance.

### Several genes express differently such as *CCND1*, *MYC* and *PGR* between MCF-7 and MCF-7/TAM cell line, of which the gene expression changed after treated with 4-OHT combined with ASA

By the gene microarray, we found that there were lots of differences of the expression of genes between MCF-7 and MCF-7/TAM cell lines. Through screening and analyzing relevant websites and database online, and with the help of previous literature [7, 12, 27, 28], we thought that there were some genes which might contribute to the phenomena of tamoxifen resistance. For example, ERα protein is the target of tamoxifen, the transcription of *PGR* is dependent on the ERα activating estrogen response element (ERE) and also code PR protein, while its level can reflect the function of ERα. Cyclin D1 protein can be regulated by ERα, and coded by *CCND1* gene, which is one of the key factors in cyclinD-CDK4/CDK6 axis and have effect on cell cycle [30]. C-myc, coded by *MYC* gene, performs multiple cellular functions such as cell proliferation, metabolism, apoptosis, growth, and differentiation [31, 32].

**Figure 4:**
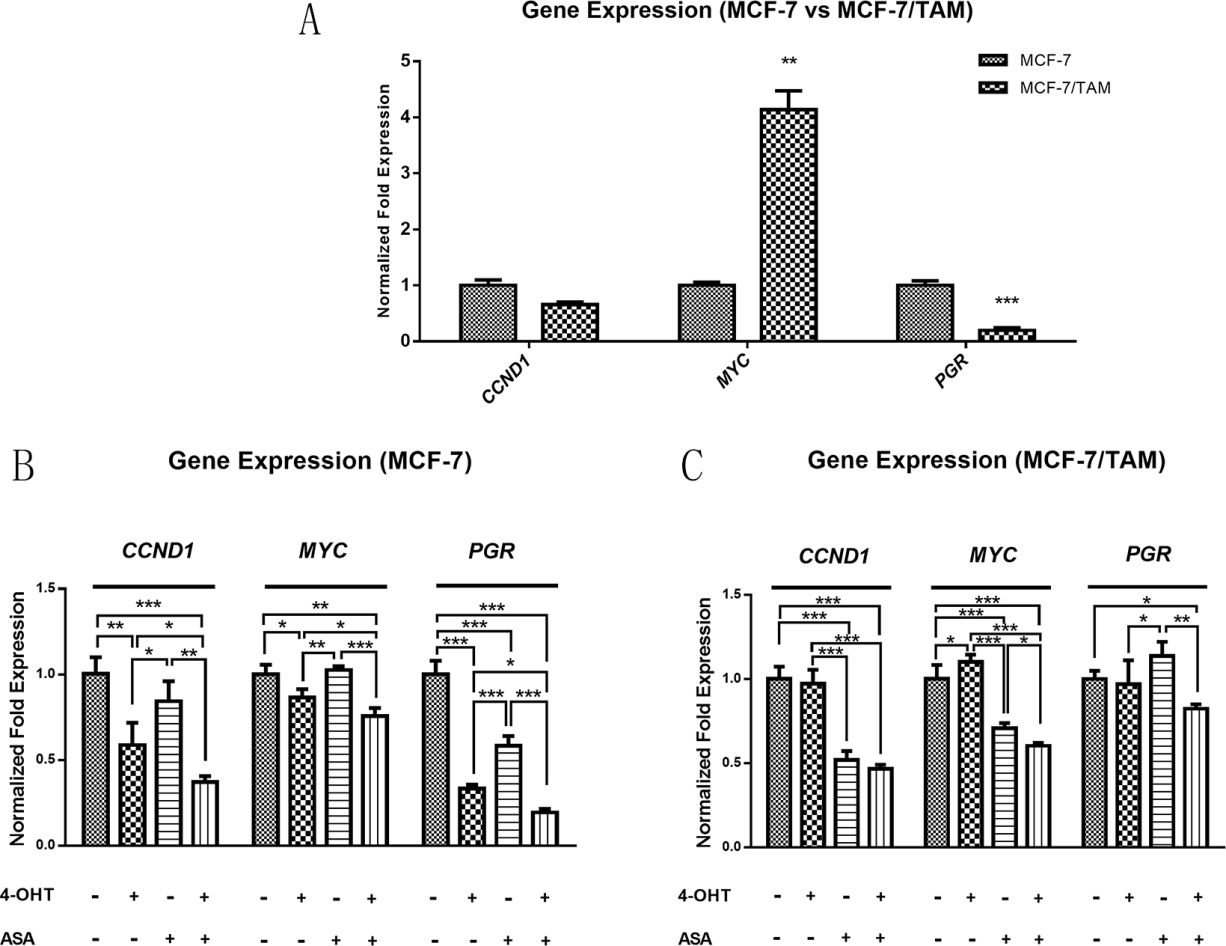
The gene expression of *CCND1, MYC* and *PGR* gene was different between MCF-7 and MCF-7/TAM cell line, which changed after treated with 4-OHT combined with ASA (**A**) There were differences of the gene expression of *CCND1, MYC* and *PGR* gene observed between MCF-7 and MCF-7/TAM cell lines (**p < 0.01, ***p < 0.001). (**B**) In MCF-7 cell line, the gene expression of *CCND1, MYC* and *PGR* was changed in ASA group, 4-OHT group and combination group compared with negative control, significant differences among these treatment groups were observed (*p < 0.05, **p < 0.01 and ***p < 0.001). (**C**) In MCF-7/TAM cell line, the gene expression of *CCND1, MYC* and *PGR* was changed only in ASA group and combination group compared with negative control, significant differences among these treatment groups were observed (*p < 0.05, **p < 0.01 and ***p < 0.001). All the experiments were repeated for at least three times. The results were presented as mean ± SEM.

As shown in Figure 4A, we found that there were differences between MCF-7 and MCF-7/TAM cell lines. The transcription of PGR had a lower expression level in MCF-7/TAM cell line. The transcription of *CCND1* gene showed no significant difference between MCF-7 and MCF-7/TAM cell lines. The expression of oncogene *MYC* was much higher in tamoxifen resistant cell line, and it might contribute to the unlimited proliferation of cancer cells. When treated with 4-OHT alone, there were differences among *CCND1, MYC* and *PGR* gene expression in MCF-7 and MCF-7/TAM cell lines. In MCF-7 cells, 4-OHT could down-regulate *CCND1*, *PGR* and *MYC* significantly, while there was no such function observed in MCF-7/TAM cell line. However, when combined 4-OHT with ASA, these genes could be down-regulated in MCF-7/TAM cells as well (Figure 4B and 4C). These changes of gene expression might reveal the sensitization mechanism of ASA.

### CyclinD1, CDK4, c-myc and PR protein levels are changed in MCF-7/TAM cells after different treatments

**Figure 5:**
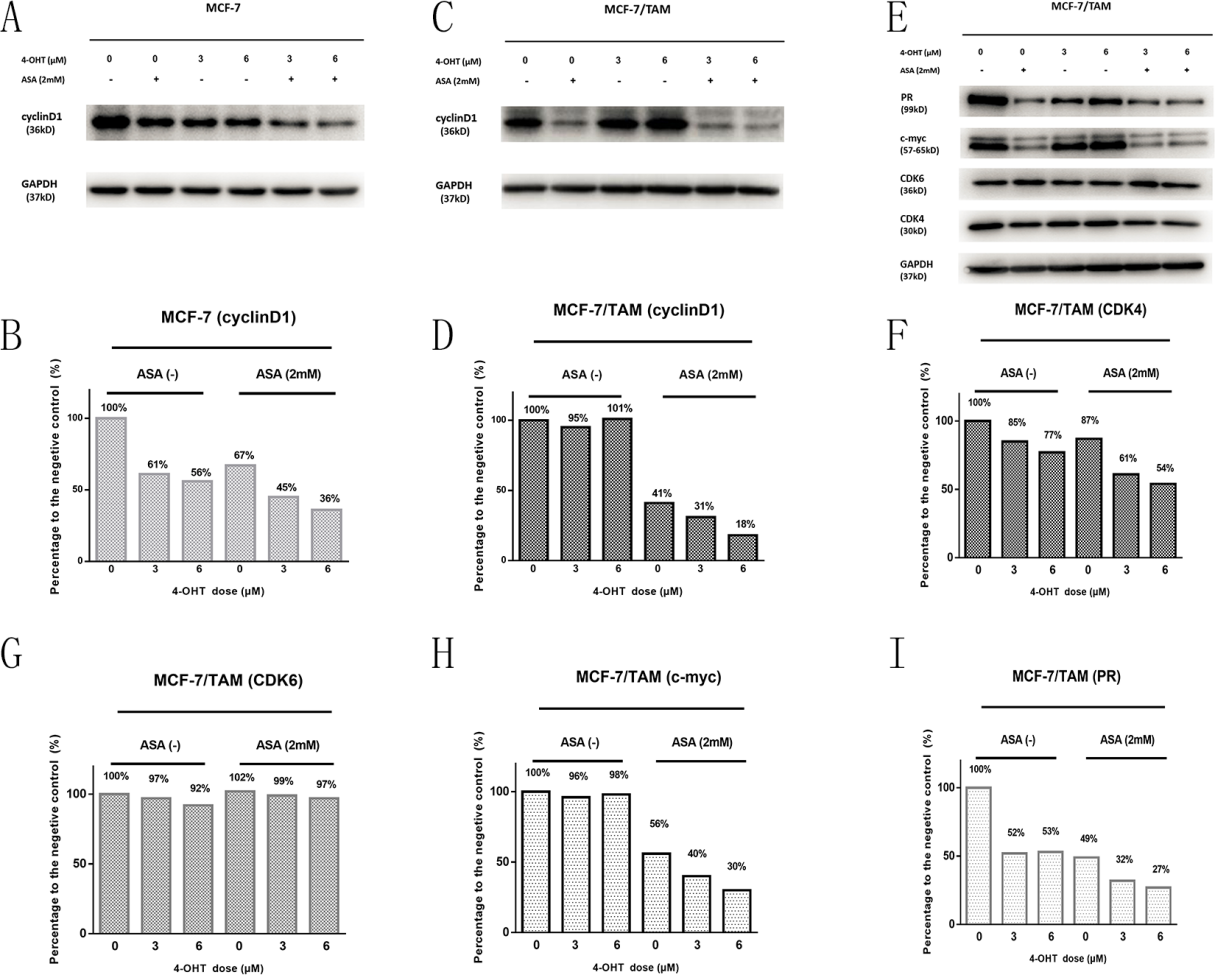
Western blotting analysis was performed to detect the changes in the protein level before and after treatment with drugs (**A**) The cyclinD1 bands of MCF-7 cell line under different drug treatments (4-OHT and ASA separate application or the two drugs combination). (**B**) Bar charts represented the cyclinD1 level compared with negative control (MCF-7). (**C**) The cyclinD1 bands of MCF-7/TAM cells under different treatments (4-OHT and ASA separate application or the two drugs combination). (**D**) Bar charts represented the cyclinD1 level compared with negative control (MCF-7/TAM). (**E**) The CDK4, CDK6, c-myc, PR bands of MCF-7/TAM cells under different drug treatments (4-OHT and ASA separate application or the two drugs combination). (**F**–**I**) Bar charts represented the CDK4, CDK6, c-myc, PR levels compared with negative control (MCF-7/TAM). All the experiments were repeated for at least three times.

To explore the molecular mechanism of aspirin and its sensitization effect on 4-OHT, western blotting was performed. As cell cycle both changed in two cell lines after drug treatment, we detected the protein level of cyclinD1, CDK4 and CDK6. CyclinD-CDK4/CDK6 complex can affect on cell cycle, which can take cell cycle from G0/G1 phase to S phase [16]. As shown in Figure 5, normally cyclinD1 protein level could be down-regulated by tamoxifen binding to ERα in MCF-7 cells. However, in MCF-7/TAM cell line, cyclinD1 protein level cannot be down-regulated by tamoxifen binding to ERα anymore. When combined ASA with 4-OHT, the inhibitory effect on cyclinD1 showed an emergence again in MCF-7/TAM cells, which enhanced by the increasing concentrations of 4-OHT. The results showed that 4-OHT could repress cyclinD1 protein levels in MCF-7 cells but not in MCF-7/TAM cells. Combination of ASA and 4-OHT could inhibit CDK4's expression in two cell lines but not CDK6, and the inhibitory effect enhanced along with the increasing concentrations of 4-OHT. This indicated that ASA overcame tamoxifen resistance by restoring 4-OHT's ability to inhibit cyclinD1-CDK4/6 complex and cell cycle in MCF-7/TAM cell line. Besides, c-myc and PR protein level were changed in MCF-7/TAM cells after different treatments, which might also contribute to overcoming tamoxifen resistance.

### The down-regulation of c-myc might be one of the reasons for aspirin to overcome tamoxifen resistance

To determine the target of aspirin, we knocked down the *MYC* gene by infecting MCF-7/TAM cells with lentivirus carrying shRNA. We successfully established the steady knock down cell line as MCF-7/TAM/sh*MYC*, and infected cells with lentivirus which carried an empty plasmid U6 as negative control. The transfer efficiency was detected by the percentage of green fluorescent, and the knockdown efficiency was determined by real-time PCR assay.

**Figure 6:**
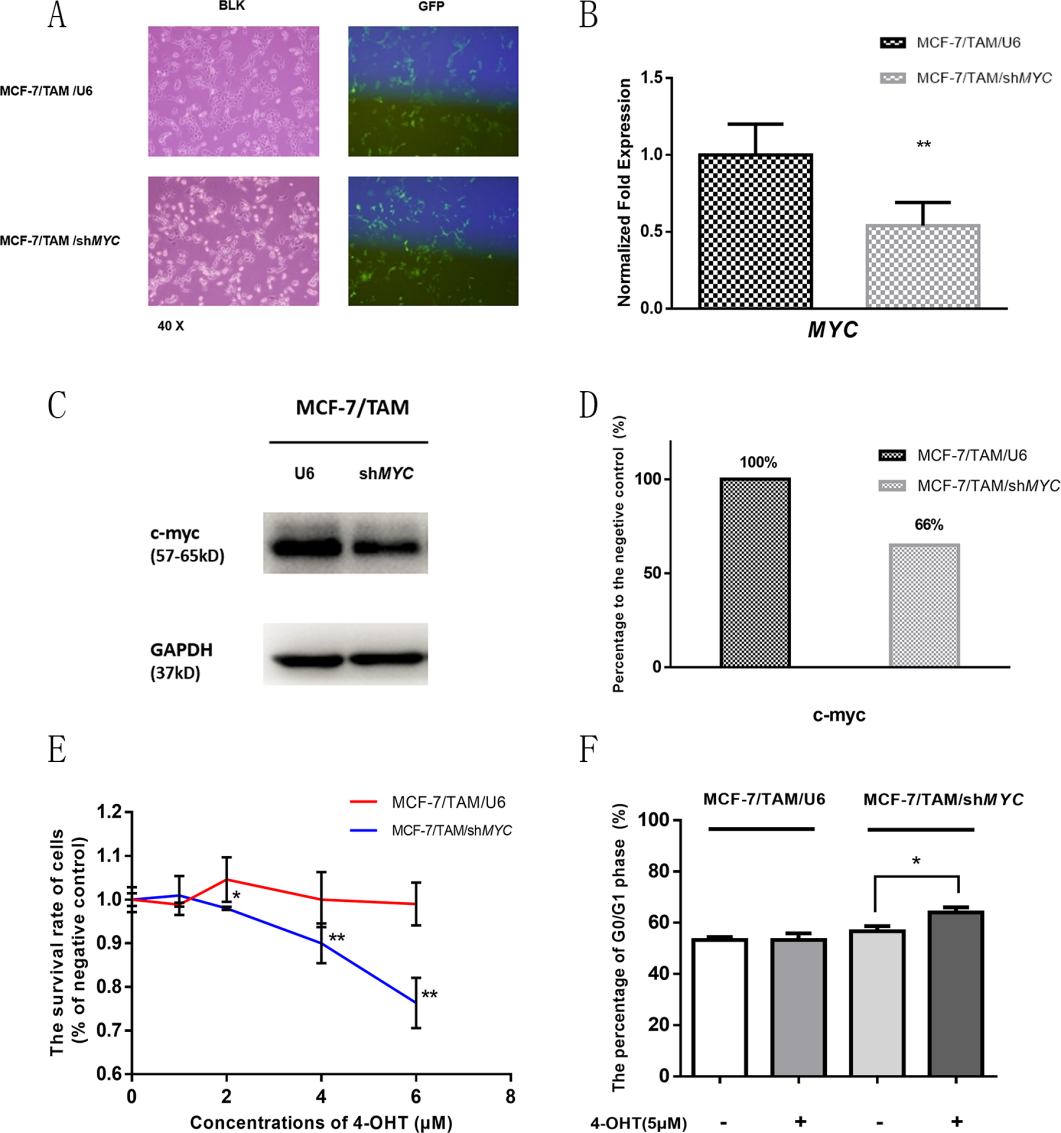
*MYC* gene was knocked down in MCF-7/TAM cell line by shRNA, and cell survival rate and cell cycle was detected after 4-OHT treatment (**A**) The transfer efficiency was detected by the percentage of green fluorescent and almost all the cells were infected by lentivirus. (**B**) The gene expression of *MYC* in MCF-7/TAM/sh*MYC* was much lower than MCF-7/TAM/U6 (**p < 0.01). (**C**) The protein bands of c-myc in two cell lines. (**D**) Bar charts represented c-myc level compared with negative control (MCF-7/TAM/U6). (**E**) The survival rate of two cell lines under different concentrations of 4-OHT showed an incremental inhibitory effect on proliferation of MCF-7/TAM/sh*MYC* cells but not on MCF-7/TAM/U6 cells (*p < 0.05 and **p < 0.01). (**F**) Bar charts represented the flow cytometry results about the percentage of G0/G1 phase. The percentage of G0/G1 phase showed a significant increase after 4-OHT treatment in MCF-7/TAM/sh*MYC* cells (*p < 0.05), but not in MCF-7/TAM/U6 cells (*p >* 0.05). All the experiments were repeated for at least three times. The results were presented as mean ± SEM.

As shown in Figure 6, almost all the cells were infected by lentivirus in MCF-7/TAM/sh*MYC* and MCF-7/TAM/U6, the gene expression of *MYC* and protein level of c-myc in MCF-7/TAM/sh*MYC* were much lower than MCF-7/TAM/U6. After treatment with different concentrations of 4-OHT for 7 days, there was an incremental inhibitory effect on the proliferation of MCF-7/TAM/sh*MYC* cells but not on control cells, and cell cycle changed as well, suggesting that cells became more sensitive to tamoxifen after knocking-down the *MYC*. Further, this indicated that *MYC* gene and c-myc protein might be one of the targets of aspirin and play important roles in overcoming tamoxifen resistance.

## DISCUSSION

Although aspirin treatment in tumor inhibition has been reported in recent years in clinical and laboratory researches [20–22, 24–26, 33], the exact molecular mechanisms are still unclear. In our study, we took ER-positive breast cancer cell lines as the research object and assessed the anti-tumor effects of aspirin on MCF-7 and MCF-7/TAM cell lines, which were sensitive and resistant to tamoxifen respectively. We found that aspirin did have an impact on ER-positive breast cancer cells’ proliferation and inhibited the process of cell cycle. Even more important, we observed the role of aspirin in partly reversing the function of tamoxifen in MCF-7/TAM cells, so we further explored the molecular mechanisms of this phenomenon, which might reflect the cause of tamoxifen resistance in ER-positive breast cancer cells.

The anti-inflammatory dose of aspirin varies from 0.5 to 2.5 mM [34], so we choose 2 mM as the concentration of ASA which is not non-specific cytotoxic, and can be used as a therapeutic dose. Aspirin inhibited the expression of c-myc protein in our study. C-myc protein levels are tightly regulated via ubiquitin-proteasome pathway in the normal cells [35–37]. But in many cancers, including breast, lung, colon and prostate cancer, c-myc is overexpressed and activated frequently [38, 39]. As a transcription factor, c-myc binds with nuclear DNA, controls nearly 15% of the expression of global genes, and performs multiple cellular functions such as cell proliferation, metabolism, apoptosis, growth, and differentiation [31, 32]. Hence, c-myc is considered as an ideal target for cancer treatment [40–42]. In this paper, compared with tamoxifen sensitive cell line MCF-7, the gene expression of *MYC* showed a higher level in tamoxifen resistant cell line MCF-7/TAM, and a higher level of c-myc protein was also observed. Since the function of c-myc had similarities with estrogen receptor, and the expression of *MYC* had a higher level in MCF-7/TAM cells, we supposed that the role of ER*α* was replaced by c-myc partially or totally. Because of this, the function of tamoxifen inhibiting cell proliferation by targeting ER*α* disappeared. Aspirin could down-regulate the c-myc protein level, and then the functions of ERα regained. Meanwhile cell proliferation and cell cycle were relied on ER*α* binding with estradiol again. In tamoxifen resistant cell line MCF-7/TAM, through knocking down the gene expression of *MYC* and suppressing the protein level of c-myc, cell proliferation and cell cycle were affected, further prompting our conclusion to be reliable.

CyclinD-CDK4/CDK6 axis has an important role in the survival and proliferation of cancer cells, which mainly takes effect through regulating the cell cycle [43]. Drugs that target this axis can be used in the treatment of cancers especially breast cancer [17, 18, 44, 45]. In tamoxifen sensitive breast cancer cell line MCF-7, 4-OHT alone could down-regulate the protein level of cyclinD1, while there was no such effect observed in tamoxifen resistant cell line MCF-7/TAM. When 4-OHT was combined with ASA, a more significant inhibitory effect of cyclinD1 protein occurred in MCF-7 cells. Besides, the cyclinD1 protein level was down-regulated in MCF-7/TAM cells at the same time. The inhibitory effect also enhanced along with the increasing concentrations of 4-OHT, which might be one of the mechanisms of aspirin to overcome the MCF-7/TAM cells’ tamoxifen resistance. Furthermore, with the recent introductions of CDK4/6 inhibitors for treatment of ER-positive advanced breast cancer [46, 47], but with the potentially unpredictable toxicity, the combined use of tamoxifen and aspirin offers a much simpler treatment. Of course, clinical trials will be need to confirm the observations reported in our studies.

**Figure 7:**
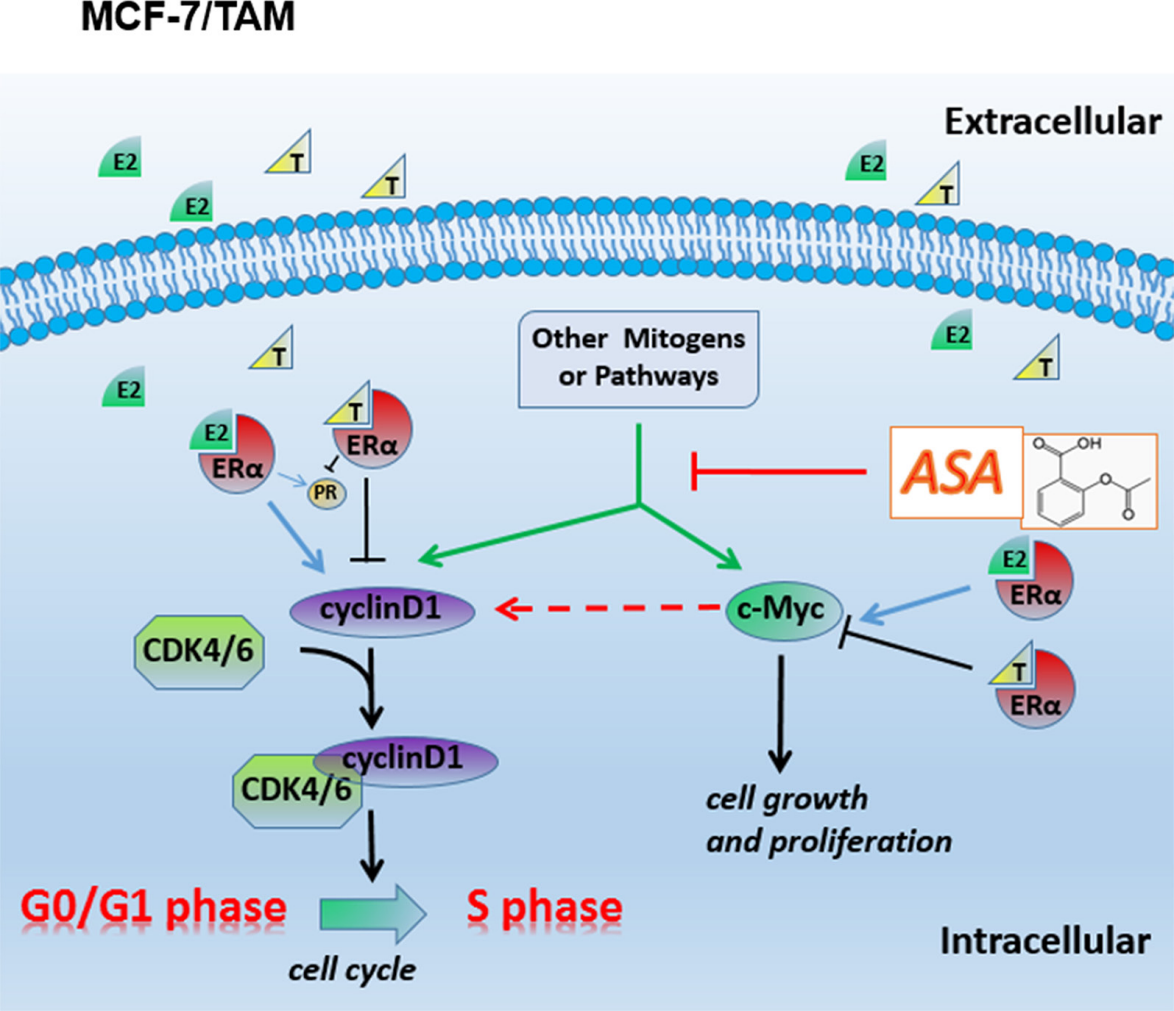
Diagramatic representation of the possible related molecular mechanism of MCF-7/TAM In the resistant cell line MCF-7/TAM, other mitogens or pathways take place E2-ER complexes’ function, so tamoxifen resistance occur. Aspirin has an effect on inhibiting c-myc and cyclin D1's expression or their upstream regulators’ activity, and partly restores the function of tamoxifen, prompting a potential role in overcoming tamoxifen resistance in ER-positive advanced breast carcinoma.

In tamoxifen resistant ER-positive breast cancer cell line, aspirin might also inhibit the upstream regulators of c-myc and cyclinD1 proteins, such as the mTOR signaling [36, 48], wnt/β-catenin [49, 50] and NF-κB pathways [51, 52]. These proteins and factors could up-regulate c-myc and cyclinD1 protein levels excessively, and these effects which were necessary for the survival of cancer cells did not depend on estradiol binding to ERα. When tamoxifen was combined with aspirin, almost all the growth signals were blocked off, and the functions of tamoxifen were regained, which reflected an effect of overcoming tamoxifen resistance in ER-positive breast cancer. Diagramatic representation of this has been shown in Figure 7. Since several large reviews were published in the latest years about tamoxifen resistance but none has mentioned aspirin or/and the pathways (c-myc and cyclins), our findings are indeed unique. Next, we are going to explore more features about c-myc protein and its regulatory mechanisms. Besides, the relationship between c-myc and cyclinD1 should also be investigated.

In conclusion, our study demonstrated that the function of aspirin inhibiting the cell cycle and proliferation of MCF-7 and MCF-7/TAM cell lines is involved in cyclin D1, c-myc and PR proteins. What's more important is that aspirin also has a potential role in overcoming tamoxifen resistance, which in turn provides a novel therapeutic strategy for ER-positive advanced breast carcinoma.

## MATERIALS AND METHODS

### Cell culture and identification

Human breast cancer cell lines MCF-7 (ER positive, estrogen dependent for growth, and anti-estrogen sensitive) and MCF-7/TAM (ER positive, estrogen independent for growth, and tamoxifen resistant, which was generated directly from MCF-7 cells by selecting against tamoxifen) were kindly presented by Dr. *Hongwu Chen* (University of California, USA) and were cultured in Dulbecco's modified Eagle medium (DMEM, Gibco, Grand Island, NY, USA) supplemented with 10% fetal bovine serum (FBS), 100 U/ml penicillin and 100 μg/ml streptomycin (Gibco, Grand Island, NY, USA) at 37°C in a 5% CO_2_ humidified incubator. Cell lines were identified by *Hua Ke Jian Lian* cell identification company (Beijing, China) in genetic analyzer to test the Amelogenin gene STR loci and gene.

### Drugs and reagents

4-hydroxy-tamoxifen (4-OHT, active product of tamoxifen), aspirin (ASA) were purchased from Sigma-Aldrich (St.Louis, MO, USA), and dissolved in Dimethyl Sulphoxide (DMSO, Sigma-Aldrich). Stock solutions were 100 mM and 1 M respectively; final DMSO concentration was under 3%. Final concentrations of 4-OHT were 1–6 μM, and final concentration of ASA was 2 mM.

### Cell growth inhibition assay

The growth inhibitory effects were measured by using a colorimetric MTS assay kit (Sigma-Aldrich). Briefly, MCF-7 and MCF-7/TAM cells were plated in 96-well plates at a density of 1 × 10^3^ cell/well, followed by an overnight incubation. Cells were then treated with increasing concentrations of 4-OHT (0–6 μM) individually or by combining with 2 mM ASA. After 7-day treatments, viable cells were quantified with MTS substrate according to the manufacturer's instructions. 450nm wave length OD values were examined by an enzyme-labeled instrument (Bio-Rad, USA). Cell viability rates were expressed as percentage of corresponding control.

### Cell cycle assay

The experiment was performed by using a cell cycle analysis kit (Beyotime Biotechnology, shanghai, China). Briefly, cells were plated in 12-well plates at a density of 2 × 10^5^ cells /well, followed by overnight incubation. Cells were then treated with 4-OHT at a concentration of 5 μM individually or by combining with 2 mM ASA. After 72 hours’ treatment, nucleus was stained by Propidium Iodide (PI) and cell cycle was examined by flow cytometry (Beckman, USA) according to the kit's manufacturer's instructions. Data was analyzed by Flow Jo 7.6 software.

### Immunofluorescent assay

Cells were plated on the glass slides. After 48 hours, cells were washed with PBS and fixed in 4% paraformaldehyde, then treated with 0.1% Triton X-100. Unspecific antigens were blocked by goat serum at room temperature and primary antibodies were incubated overnight at 4°C. The CY3-secondary antibodies (Abcam, USA) were incubated and FITC-phalloidin solution was used bind F-actin. Cell nucleus was stained by DAPI working solution. Finally, cells were photographed using LSM 510 META confocal microscope (Carl Zeiss, Germany).

### Quantitative real-time RT-PCR assay

Total RNA was extracted by using TRIzol reagent (Sigma-Aldrich), and cDNA was synthesized from total RNA using a Reverse Transcript Kit (Promega, USA). Relative quantitation of mRNA expression was achieved using real-time PCR (CFX Real-Time PCR system; Bio-Rad USA). The SYBR Green Master Mix (Promega) was used according to the manufacturer's instructions and the level of gene expression was measured by calculating 2^−ΔΔ^Ct. The sequences of primer for qPCR were as follows: *ESR1-forward, TCCAAACCCATCGTCAGTGT. ESR1-reverse, TGAATGCAAAGGGGTCTGTGT. CCND1-forward, AAT GACCCCGCACGATTTCA. CCND1-reverse, TGAGGCG GTAGTAGGACAGG. MYC-forward, CTTGTTGCGGAA ACGACGAG. MYC-reverse, ACTCAGCCAAGGTTGT GAGG. PGR-forward, AGGTCTACCCGCCCTATCTC. PGR-reverse, AGTAGTTGTGCTGCCCTTCC. GAPDH-forward, CCCACTCCTCCACCTTTGAC. GAPDH-reverse, TGTTGCTGTAGCCAAATTCGTT*.

### Western blot analysis

After treatment with drugs, whole-cell lysates (20μg) were separated by 10% SDS-PAGE, and then proteins were transferred to PVDF membranes (Pall Corporation, Life Sciences). Membranes were blocked by 3% BSA and probed with antibodies. The quantification of protein level was performed by densitometric scanning (Tanon-5500, Shanghai, China) and then was normalized to intensity with GAPDH. The results of Western blotting were analyzed by *ImageJ* software. Antibodies for Western blot were as follows: rabbit c-myc and rabbit PR primary antibodies were purchased from Abcam (USA) at 1:1000 dilution in 5% BSA. Rabbit cyclinD1, rabbit GAPDH primary antibodies and mouse anti-rabbit HRP antibodies were purchased from Cell Signaling Technology (USA) at 1:2000 dilution in 5% BSA.

### Gene knocking down

Complementary oligos (synthesized by Invitrogen, USA) containing an *MYC* directed shRNA sequence 5′-GATCTGGAGATGATGACCGAGTTACCTCGAGGT AACTCGGTCATCATCTCCATTTTTG-3′ were cloned into the BamHI / MluI site of pLent-U6-GFP-Puro vector following the manufacturer's cloning protocol (New England Biolabs, USA). Plasmid was isolated and purified using Plasmid Mini Kits I (Omega, USA), then plasmid was transformed into DH5α E. coli competent cells. Lentivirus was packaged using 293T cells, and then transfected into MCF-7/TAM cells following the relevant protocols. Empty plasmid was used as negative control group at the same time. Cell lines MCF-7/TAM/shMYC and MCF-7/TAM/U6 were established from MCF-7/TAM by the selection of puromycin (Sigma-Aldrich). The transfer efficiency was detected by the percentage of green fluorescent and the gene knocking down efficiency was detected by RT-qPCR assay.

### Statistical analysis

Data was presented as mean ± SEM. Each experiment was performed independently for at least 3 times. SPSS 19.0 statistical software package (IBM, USA) was applied to analyze data. Student's *t* test was used to compare two group-design experiments. One-way ANOVA was used to compare the effects of multiple group-design experiments, and LSD, SNK were used as post hoc test. *P <* 0.05 was considered to be statistically significant.
